# Evaluation of Iodinated Contrast Media Use in Abdominal CT Scans in Cancer Assessments: A Cross-Sectional Study in Lomé (Togo)

**DOI:** 10.1155/2023/8296467

**Published:** 2023-01-05

**Authors:** Pihou Gbande, Bidamin N'timon, Dermane Alassani Tchakpedeou, Mazamaesso Tchaou, Kokou Adambounou, Lantam Sonhaye, Lama Kegdigoma Agoda-Koussema, Komlanvi Adjenou

**Affiliations:** ^1^Sokodé Regional Hospital Centre, Department of Radiology and Medical Imaging, Sokodé, Togo; ^2^Campus University Hospital Centre, Department of Radiology and Medical Imaging, Lomé, Togo; ^3^Kara University Hospital Center, Department of Radiology and Medical Imaging, Kara, Togo; ^4^Sylvanus Olympio University Hospital Center, Department of Radiology and Medical Imaging, Lomé, Togo

## Abstract

**Background:**

There is great variability between centers regarding contrast injection protocols. They should only be injected if they can provide useful information for diagnosis with the necessary and sufficient quantity of iodine. We wanted to know through this study if the use of iodinated contrast media is optimised in abdominal CT scans performed for cancer assessment in Lomé.

**Materials and Methods:**

It was a cross-sectional, descriptive, and analytical study with a prospective collection over a period of 6 months in three CT units in Lomé. It involved abdominal CT scans performed for oncological evaluation. Data were reported as the mean ± standard deviation. The Pearson correlation coefficient, ANOVA, chi-square, and the Fisher test were used.

**Results:**

A total of 218 examinations were recorded. The female sex represented 56.88% of the patients. The mean age was 50.92 ± 15.78 years. The mean weight was 70.46 ± 15.23 kg. The mean BMI was 24.91 ± 5.32 kg/m^2^. The examinations were performed with a voltage of 120 kV in 195 cases (89.45%). The mean dose of injected iodine was 0.42 ± 0.09 gI/kg with a dose of 0.40 gI/kg at 80 kV and 0.45 gI/kg at 130 kV. The mean injection rate was 2.90 ± 0.34 mL/s. The mean injected volume was 83.19 ± 7.29 mL. The mean duration of the injection was 30.60 ± 7.39 s. The mean iodine delivery rate was 0.98 ± 0.17 gI/s. There was no saline injection in 152 cases (69.72%). Liver contrast enhancement was satisfactory in 94.5% of cases. There was a strong negative linear correlation between the dose of injected iodine and weight.

**Conclusions:**

Optimization guidelines for the use of iodinated contrast media are not always applied. Therefore, monitoring and benchmarking programmes for iodinated contrast injection protocols that involve all radiology personnel should be implemented.

## 1. Introduction

In medical imaging, a contrast agent is a substance that artificially increases contrast to visualize an anatomical structure or pathological structure that is naturally low or noncontrast and which would therefore be difficult to distinguish from neighboring tissue [1]. The use of contrast media in CT scans has been the subject of ongoing research in recent years [2–5]. Although the injection of an iodinated contrast medium during a CT examination is very frequent in the clinical practice of a radiologist, there is great variability between centers regarding the injection protocols used. Factors that affect contrast enhancement in CT are the concentration and dose of iodinated contrast medium (ICM), the injection rate, the duration of acquisition, the flush of saline, and cardiac output [6, 7]. Dose of ICM, expressed in gI/kg, is one of the most important factors that determine contrast enhancement of the liver parenchyma [8].

Justification and optimization are, as with ionizing radiation, the basis for the use of iodinated contrast media. They should only be injected if they can provide useful information for diagnosis with the necessary and sufficient quantity of iodine. Abdominal computed tomography is one of the most frequent procedures in a radiologist's clinical practice. However, liver CT imaging protocols are still not sufficiently standardized. Taking into account that a fixed amount of iodine is to be administered to patients, which is still a common practice [9], some patients may receive an unnecessary high dose, while others may receive a suboptimal dose. To overcome this drawback, the working group of the Interdisciplinary Committee for Research and Work on Contrast Agents in Imaging (CIRTACI) in France proposed to start optimizing injection protocols with a maximum dose of 0.5 gI/kg [10].

In Togo, for several years, the requirement for quality of services rendered to the client has been increasingly at the heart of the concerns of public authorities. To contribute to good practices in the use of contrast media in our facilities, this study was carried out with the objective of evaluating protocols in the use of iodinated contrast media in abdominal CT scans performed for cancer assessment.

## 2. Materials and Methods

This was a cross-sectional, descriptive, and analytical study with a prospective collection over a period of 6 months (from April 1 to September 30, 2022). It took place in three hospitals in the city of Lomé, Togo. These were Campus University Hospital, the “Autel d'Elie” clinic, and the “le Coeur” polyclinic. In this study, all patients referred to the radiology departments of these hospitals for abdominal CT (for initial cancer diagnosis) or thoracoabdominopelvic CT (TAP CT) for cancer staging during the study period. CT scans performed on an emergency basis were not included in this study.

The examinations were performed at Campus University Hospital and at the “Autel d'Eli” clinic with a 16-row CT scan and at the “le Coeur” polyclinic with a 64-row CT scan. Iterative image reconstruction algorithms were not available on all CT scanners used. An arterial phase was performed before the portal venous phase for all CT scans. The variables studied were as follows:

  Patients' characteristics: age, sex, weight, height, BMI, and location of the primary cancer in the patient
  Acquisition and iodinated contrast media protocols: kV, mA, concentration, volume, injection rate, duration of ICM injection, and contrast enhancement of the contrast of the liver parenchyma

We considered patients aged 15 years or younger as a paediatric population.

According to the WHO international classification of body mass index (BMI) [11], patients were considered underweight when the BMI was less than 18.5 kg/m^2^, normal weight when it was between 18.5 kg/m^2^ and 25 kg/m^2^, overweight when it was between 25 kg/m^2^ and 30 kg/m^2^, and obese when it was greater than 30 kg/m^2^.

**Figure 1:**
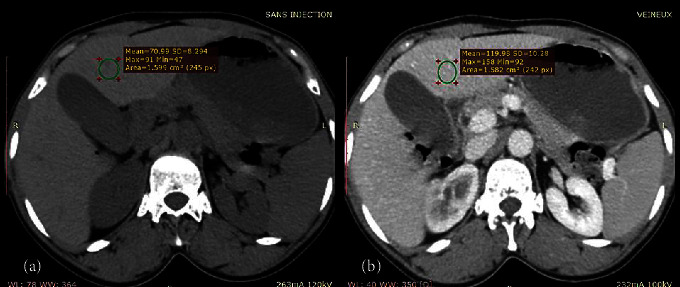
Liver contrast enhancement measurement: noncontrast image (a); portal venous phase image (b).

An enhancement of at least 250 HU of the abdominal aorta was considered acceptable for the arterial phase. Liver parenchymal contrast enhancement was calculated as the difference between the density of the liver at the portal venous phase and the noncontrast phase measured using a ROI placed on segment IV at the same slice level (Figure 1). The area of the ROI was about 1–2 cm^2^. All ROIs avoid inclusion of the venous and arterial vessels. The enhancement was said to be insufficient if it was less than 50 HU, satisfactory if it was between 50 and 60 HU, and good if it was greater than 60 HU [8, 10, 12].

The data were processed using EPI Info 7.1.3.3 software. A significance level of 5% was established for the statistical tests. The chi-square test was used to analyze and compare the data of the categorical variable groups. Fisher and Student's *t*-test was used to compare the data of the groups of quantitative variables, and the Pearson coefficient was used to show the correlation between these quantitative variables.

## 3. Results

### 3.1. Patients' Characteristics

A total of 218 patients were enrolled in this study. The female sex represented 124 patients (56.88%), and the male sex represented 94 patients (43.12%) with a sex ratio of 1.3. The mean age was 50.92 ± 15.78 with extremes of 6 and 85 years. The mean weight of the patients was 70.46 ± 15.23 kg, and the mean height was 1.67 ± 0.08 m. The mean BMI was 24.91 ± 5.32 kg/m^2^. Most of the patients had normal weight (*n* = 114; 52.29%). The distribution of patients by BMI is recorded in Table 1.

Digestive cancers were the most observed, representing 144 patients, or 66.05% with 52 cases of liver cancers (23.85%), followed by lung cancers (33 cases; 15.14%) and then breast cancer (26 cases; 11.93%) according to Table 2.

### 3.2. Protocols for the Acquisition and Use of Iodinated Contrast Media

We recorded 123 TAP CT scans, or 56.42%, and 95 abdominal CT scans, or 43.58%. One hundred ninety-six CT scans (89.91%) were performed on a 16-row scanner, and 22 scans (10.09%) were performed on a 64-row scanner. Most of the CT scans were performed with a voltage of 120 kV (195 exams; 89.45%). The mean tube current-time product was 253.09 ± 72.53 mAs. The mean dose of injected iodine was 0.42 ± 0.09 gI/kg. The contrast medium most used had a concentration of 350 mgI/mL, 177 cases (81.19%). The mean injection rate was 2.90 ± 0.34 mL/s. The majority of CT scans were performed with an injection rate of 3 mL/s (156 cases; 71.56%). Four CT scans were performed with an injection rate of less than 2 mL/s (1.84%) (Table 3). The mean volume of ICM was 83.19 ± 7.29 mL (ranging from 20 to 95 mL). The mean ICM injection duration was 30.60 ± 7.39 s. The majority of the CT scans (*n* = 193; 88.54%) had an injection duration of less than 30 s, and 25 CT scans (11.47%) had an injection duration of more than 30 s. The mean iodine delivery rate was 0.98 ± 0.17 gI/s (ranging from 0.34 to 1.26 gI/s) for all CT scans. The mean acquisition duration was 13.43 ± 5.21 s. It was 12.70 ± 5 s for abdominal CTs and 13.99 ± 5.32 s for TAP CTs.

Only 66 CT scans, or 30.28%, had received a saline injection immediately after the contrast medium. Thirty-six CT scans (16.51%) had insufficient arterial phase. Twelve CT scans (5.50%) had insufficient liver contrast enhancement; 33 CT scans (15.14%) had satisfactory liver contrast enhancement, and 173 CT scans (79.36%) had good liver contrast enhancement. The mean liver contrast enhancement was 78.88 ± 17.42 HU. The dose of injected iodine was 0.40 gI/kg at 80 kV and 0.45 gI/kg at 130 kV (*p* = 0.60). On the other hand, liver contrast enhancement decreased with voltage (*p* = 0.12). There was a strong negative linear correlation between BMI and iodine dose (*r* = −0.78; *p* < 0.00001) as well as between weight and iodine dose (*r* = −0.72; *p* < 0.00001). The injected iodine dose was greater when the injection duration was greater than 30 s (*p* = 0.002). Liver contrast enhancement was significant when the injection duration was less than 30 s (*p* = 0.04). There was no relationship between the iodined contrast injection rate and hepatic enhancement (*p* = 0.69).  Table 4 shows the mean dose of iodine and liver contrast enhancement according to the different parameters.

The contrast medium injected volume was greater when the concentration was high, and this relationship was close to significance (*p* = 0.06). The dose of injected iodine was low with a lower ICM concentration (*p* < 0.00001) as shown in Table 5.

## 4. Discussion

A total of 218 patients were enrolled in this study. Female sex was predominant with a sex ratio of 1.3. Amégbor et al., in 2011, in an epidemiological study of 5251 cancer cases in Togo found a similar result with a sex ratio of 1.11 [13]. Also, Garba et al. [14], in Niger, reported a female predominance. However, Darré et al. in 2016 reported a male predominance in their series with a sex ratio of 0.7 [15]. The mean age was 50.9 years. This result is close to that of Darré et al., who found a mean age of 50.4 years [15]. Amégbor et al. found a slightly lower age of 45.3 years [13]. Similarly, Effi et al. [16] in Côte d'Ivoire and Garba et al., [14] in Niger reported an average age of 49.2 and 43 years, respectively.

The majority of the CT scans were performed with a voltage of 120 kV, and the average tube current-time product was 253.09 ± 72.53 mAs. Adambounou et al. in 2021 in Togo reported that the acquisition parameters (tube current-time product and voltage) were almost identical for all types of CT scans, with more than 4/5 of the examinations performed at 120 kV [17]. In a study by Mbozo'o Mvondo et al. in Cameroon, the average kilovoltages and tube current time product were, respectively, 90 kV and 112.5 mA for abdominal-pelvic scans [18]. Voltage is a parameter that influences the penetration of the X-ray beam into the patient. It expresses the X-ray photon rate, which directly influences the dose delivered to the patient as well as the signal-to-noise ratio [1]. The voltage must be adapted to the thickness of the body examined. For radiation protection, it is necessary to reduce the voltage as soon as possible (for children and thin people). Going from 120 to 80 kV theoretically allows for a 65% reduction in CTDI_vol_ [19]. The current-time product also has an impact on the quantity of X-rays emitted during an acquisition [1]. Unlike the voltage, this parameter is hardly ever adjusted directly by the user. The user will mainly adjust the milliamperage (mA) which determines the quantity of electrons. Currently, the milliamperage is often automatically adjusted by the device using dose optimization software.

In this study, the contrast medium commonly used had a concentration of 350 mgI/mL (81.19%), and the average dose of injected iodine was 0.42 ± 0.09 gI/kg. Zanardo et al. found a dose of approximately 0.46 gI/kg [20]. These values are well below the data which recommends an optimal dose of 0.6 gI/kg [21–23]. It is the iodine dose that determines the enhancement of the organs examined. In oncology, and in particular for thoracoabdominopelvic CT scans, the aim should be to obtain an enhancement of about 50 HU of the liver at the portal phase (compared to a noncontrast liver). If the scan is injected at the outset, the aim should be to achieve a liver density of 110 HU or more [10]. The mean liver contrast enhancement in this study was 78.88 ± 17.42 HU. Most of the examinations had a liver contrast enhancement greater than 50 HU. The mean volume and the mean injection rate were low in this study, corresponding to 83.19 ± 7.29 mL and 2.90 ± 0.34 mL/s, respectively. Lauretti et al., in an intrainstitutional audit, reported a mean volume of 93.73 ± 17.58 mL and a flow rate of 3.53 ± 0.89 mL/s [24]. Paradoxically, we observed in this study that the higher the ICM concentration, the greater the volume injected. This shows that the concentration of the ICM was not taken into account when calculating the volume of the medium to be injected. It could be due to the lack of radiology personnel's care to optimization and the lack of auditing on CT protocols. In principle, the volume of the iodinated contrast medium to be injected should take into account the concentration by injecting the same dose of iodine for patients of the same weight [25]. The implementation of an automated iodinated contrast media management system, as demonstrated in the study by Lauretti et al., could assist in the effective management by improving and harmonising the quality of injection protocols [24].

According to the CIRTACI working group, the ICM injection rate is adapted according to the calculated volume to have a maximum injection duration of 30 s [10]. In our series, the mean injection rate was 2.90 ± 0.34 ml/s and the mean ICM injection duration was 30.60 ± 7.39 s with 11.47% of examinations having an injection duration of more than 30 s. In reality, the injection must be completed before the end of image acquisition especially for the arterial phase [10]. Increasing the injection duration alters the quality of the enhancement of the parenchyma and increases the dose of iodine to be injected. This was observed in this study where the dose of iodine injected was higher when the injection time was longer than 30 s, thus requiring an unnecessary additional dose of iodine. It is reported that of the technical parameters used in the injection protocol for multiphase CT of the liver, the fixation of the ICM duration of the injection is the most important factor for the accurate estimation of the delay for each acquisition phase [23].

Only 30.28% had received a saline injection immediately after the contrast medium. Rinsing with saline (30–40 ml of saline is sufficient) to remove the contrast medium from the tube and the proximal venous system optimises the enhancement of the parenchyma [26]. This optimization factor requires adapted equipment (dual barrel injectors). This equipment was only available in one of the three centers. In this study, we found that the higher the voltage, the more the iodine used. The dose of iodine injected was 0.40 gI/kg at 80 kV and 0.45 gI/kg at 130 kV. In contrast, liver contrast enhancement decreased as voltage increased. Studies have shown that reducing the kilovoltage allows a reduction in the X-ray dose and the useful quantity of the contrast medium [22, 27, 28]. Indeed, the effectiveness in terms of contrast medium attenuation is then identical for a lower quantity of iodine, while reducing the X-ray exposure dose. Schematically, a 20 kV reduction reduces the X-ray dose and the iodine dose by 20% [10]. Therefore, it is desirable that at 100 kV, the initial dose of the iodine dose to be used is 0.4 g I/kg and 0.32 g I/kg at 80 kV [10, 29, 30].

There was a strong negative linear correlation between the dose of injected iodine and patient weight. Obese patients received a lower dose of iodine, whereas underweight patients received a dose above the recommended range. Although there are no recommendations for obese patients, radiologists had subjectively reduced the iodine dose in these patients as a precautionary measure. An obese patient has a high proportion of body fat, a proportionally small blood volume compared to total body weight, and a small and well-perfused extracellular compartment. Thus, using a linear iodine dose-weight relationship overestimates the iodine dose. Therefore, other more relevant parameters, such as body surface, have been proposed to account for obesity and avoid iodine overdose [10, 31, 32].

## 5. Conclusions

Oncology CT scans are becoming increasingly common in our radiological practice. Digestive cancers are the most frequently encountered. Most of the patients were of normal weight. The majority of the CT scans allowed sufficient enhancement of the parenchyma, but the measures for optimizing the injection of iodinated contrast media are not always applied. These measures mainly concern the injection of serum saline immediately after the contrast medium, the reduction of the kilovoltage, the fixing of the duration of injection, and the consideration of the concentration of the contrast medium when calculating the volume necessary to inject. Monitoring and benchmarking programmes for iodinated contrast injection protocols involving all radiology staff should therefore be implemented.

## Figures and Tables

**Table 1:** Distribution of patients by BMI.

BMI	Number	Percentage
Underweight	15	6.88
Normal weight	114	52.29
Overweight	58	26.61
Obese	31	14.22
Total	218	100.00

**Table 2:** Distribution of patients by the type of primary cancer.

Primary cancer	Number	Percentage
Brain	1	0.46
Digestive tract	92	42.20
Liver	52	23.85
Bones	4	1.83
Lung	33	15.14
Prostate	4	1.83
Breast	26	11.93
Bladder	6	2.75
Total	218	100.00

**Table 3:** Acquisition and ICM use parameters.

Items	Number	Percentage
Voltage		
80 kV	2	0.92
120 kV	195	89.45
130 kV	21	9.63
Total	218	100.00
ICP concentration		
300 mgI/ml	5	2.29
350 mgI/ml	177	81.19
370 mgI/ml	36	16.51
Total	218	100.00
Injection rate		
1 mL/s	2	0.92
1.5 mL/s	2	0.92
2 mL/s	2	0.92
2.5 mL/s	40	18.35
3 mL/s	156	71.56
3.5 mL/s	16	7.34
Total	218	100.00

**Table 4:** Mean iodine dose and liver contrast enhancement according to the different parameters.

Items	Iodine dose	Liver contrast enhancement
Voltage		
80 kV	0.40 ± 0.03	80.50 ± 3.53
120 kV	0.42 ± 0.08	79.64 ± 17.41
130 kV	0.45 ± 0.11	71.47 ± 16.99
BMI		
Underweight	0.58 ± 0.09	85.93 ± 16.52
Normal weight	0.45 ± 0.07	79.10 ± 19.10
Overweight	0.38 ± 0.03	80.39 ± 13.91
Obese	0.32 ± 0.05	71.83 ± 15.65
Injection duration		
<30 s	0.42 ± 0.08	79.74 ± 17.35
>30 s	0.47 ± 0.10	72.28 ± 16.88
Injection rate		
1 mL/s	0.40 ± 0.03	82.50 ± 3.53
1.5 mL/s	0.48 ± 0	90.00 ± 12.72
2 mL/s	0.42 ± 0	50.50 ± 0.70
2.5 mL/s	0.42 ± 0.10	74.40 ± 16.63
3 mL/s	0.43 ± 0.09	79.98 ± 17.38
3.5 mL/s	0.41 ± 0.03	81.06 ± 18.45

**Table 5:** Concentration and volume of injected ICM.

ICM concentration (mgI/mL)	Injected volume	Iodine dose
300	81.00 ± 2.23	0.34 ± 0.02
350	82.94 ± 7.97	0.42 ± 0.08
370	84.72 ± 2.37	0.47 ± 0.10

## Data Availability

The data used to support the findings of this study are included within the article.
